# Hospital and Health News

**Published:** 1923-03

**Authors:** 


### HOSPITAL AND HEALTH NEWS.

### Presentation to a Hospital Secretary.

Mr. E. Weldon, honorary secretary of the Clacton and District Cottage Hospital, has been presented with a gold watch and an illuminated address in recognition of his services to the institution.

### Royal Northern Hospital.

Lady Newnes, Lady Sargant, and others are arranging a dance at the Hyde Park Hotel, on the Royal Wedding Night (April 26), in aid of the new X-ray Department of the Royal Northern Hospital.

### A Prize Essay.

Dr. Wynne, Medical Officer of Health for Sheffield, has won the hundred-guinea prize offered by the National Baby Week Council for an essay on the problem of excessive mortality among boy babies.

### Beds for Convalescents.

A retired doctor's wife has two beds for convalescent destitute children in the Birmingham Children's Hospital. She will pay all expenses and take each child for three months.

### Hospital Amalgamation.

The recent amalgamation of the Alexandra Hospital for the treatment of hip disease and St. Bartholomew'sHospital provides a large London Hospital with a home of its own for such cases for the first time. Thus the Kettlewell Convalescent Home at Swanley becomes the Tuberculosis Home of St. Bartholomew's Hospital.

### C.M.B. for Scotland.

Dr. James Haig Ferguson has been unanimously re-elected Chairman of the Central Midwives' Board for Scotland. Dr. Michael Dewar was elected Deputy Chairman, and Sir Archibald Buchan-Hepburn, Bt., Convener of the Finance Committee.

### Wright's Sash Chains.

A considerable saving of broken window frames, glass and cords and of damaged woodwork and paint can be made by the fitting of Wright's sash chains, which are exceedingly strong, safe and permanent and can be fixed at a very low cost. "Wright" chains are in use in many Government departments, in the chief London hospitals and in many county asylums. Catalogues and price list can be obtained from Messrs. Charles Wright, Ltd., Edgware.

### R.A.M.C. Commissions.

The following were the successful candidates for Commissions in the Royal Army Medical Corps at the competition held in London in January:—H. A. Gilkes, M.B., Ch.B. (Oxford), Oxford University, St. Bartholomew's Hospital; R. Murphy, B.A., M.B., B.Ch., B.A.O. (Dublin), Trinity College, Dublin; F. C. H. Sergeant, M.B., Ch.B. (Liverpool), Liverpool University; W. L. S. Cox, M.R.C.S. (England), L.R.C.P. (London), University College Hospital.

### National Baby Week Council.

Since its foundation in 1917 the National Baby Week Council has arranged over two thousand Baby Week celebrations, formed over one thousand local committees, held three thousand meetings for mothers, schools, &c., and its three hundred Child Welfare Exhibitions have been attended by over a million people. It may, therefore, fairly claim a share in the credit for the great reduction in infant mortality and children's damage rate in this country. Its life is, however, imperilled by lack of funds, and without increased support the work can neither be sustained nor extended. The headquarters of the Council are at 117 Piccadilly, W.1.

### Examination for Health Visitors.

The regulations for admission to the examination of the Royal Sanitary Institute have been extended to include the qualifications for health visitors named in the memorandum issued by the Ministry of Health in July, 1922. From January, 1923, all candidates for the Institute's certificate will be required to have: Training in the duties of a nurse at a recognised hospital training school for a period of not less than three years; with the certificate of the Central Midwives' Board or other equivalent qualification in midwifery; and also attendance at a course of training approved by the Institute.

### Wireless Installation for a Hospital.

Through the generosity of friends a complete wireless installation, with loud speakers, has been provided for the in-patients, on both the male and female floors of the Westminster Ophthalmic Hospital, many of whom are blind, and consequently find the time tedious. The nursing staff have also a "listening in" set for their own use, which has been given by the Marconi Company.

### St. Mark's Hospital for Cancer, &c.

At the 87th annual general meeting of the Governors, reference was made to a much-needed extension of the hospital premises on vacant land adjoining the present building. This extension, which would cost about £10,000, would provide accommodation for another 21 beds and also a new out-patients' department, as well as new pathological and X-rays departments. During 1922, in which there had been a decrease of £431 in ordinary donations, the hospital had admitted 650 in-patients, of whom 581 had been cured; 3,815 attendances had been made by 1,130 new out-patients during the same period.

### League of Nations and Public Health.

The Health Committee of the League has decided that the next interchange of public health officials, organised by the League and financed by the Rockefeller Foundation, shall take place in England. The course will probably last three months and will consist of lectures and visits to hospitals, asylums, bacteriological laboratories, &c. Those taking part will also be sent to various parts of the country to be attached to a branch of the public health services. A similar course is to take place in Austria, and the Italian Government has agreed to a special course on methods of combating malaria to be organised in Italy.

### Guildford Poor Law Infirmary.

The frequent changes in the nursing staff of the infirmary under the Guildford Guardians have led the establishment Committee to investigate and to issue a report. This finds that the superintendent nurse is conscientious and experienced, that the food, the nurses' home and their sleeping quarters are excellent, but that the work is very hard, the staff insufficient, and that an extension of late passes for the theatre and social engagements is desired. In regard to the last three points the board is trying to improve the nurses' conditions. In the main the Committee is satisfied with the organisation of the infirmary both from the point of view of the patients and of the staff.

### New Laboratories for "Bart's."

The medical college of St. Bartholomew's Hospital, which is now attended by some eight hundred students, and is probably the largest medical educational institution in England, is shortly to be extended. The college authorities have acquired a block of modern premises in Giltspur Street, facing the main hospital, for the new laboratories, lecture rooms, &c. The negotiations have been carried through by Messrs. Knight, Frank & Rutley, in conjunction with Messrs. Leopold Farmer & Sons.

### Examinations under the Dentists' Act.

The third series of the Prescribed Examination under the Dentists' Act, 1921, will be held during April; the closing dates for entries are as follows:—For examination in London, March 6; for examination in Manchester, March 21; and for examination in Edinburgh, March 30. Notices will shortly be sent to all those whose names are on the list of candidates. Those whose names are entered on the list of practitioners of less than five years' standing and are intending to pass the Prescribed Examination should bear in mind that there will only be two more opportunities for them to present themselves, April and July of this year. All communications should be addressed to the Secretary for Examinations, 44 Hallam Street, W.1.

### Infant Care Lectures at Northampton.

A course of lectures on Infant Care for health visitors, nurses, infant welfare workers, &c., will be held at the Lower Assembly Room, Town Hall, Northampton, on Mondays, from Feb. 26 to March 26, inclusive. Two lectures will be given each day from 11.45 a.m. to 12.45 p.m., and from 1.45 to 2.45 p.m. Further particulars can be obtained from the Secretary, National Association for the Prevention of Infant Mortality, Carnegie House, 117 Piccadilly, W.1.

### Typhoid Bacilli in Mussels.

In consequence of the prevalence of infectious disease in Bradford seven samples of mussels were submitted for bacteriological examination by the City Bacteriologist. Five of the seven samples were found to show distinct evidence of contamination by typhoid bacillus. It has accordingly been decided, in accordance with Article 3 of the Public Health (Shell Fish) Regulations, 1915, to make representations to the local authority concerned (in this case the Preston Port Authority). The contamination is supposed to be due to sewage finding its way to the mussel "layings."

### Approved Societies Help Manchester Infirmary.

For the first time contributions from Approved Societies appear in the accounts included in the report for the past year of the Manchester Royal Infirmary. The board records its thanks not only for the grant, but also for the courtesy of the officials which rendered the collection of this large sum (£4,128 in nine months) "a comparatively easy matter." The total expenditure for the year was £112,000, and the income £12,000 short of this amount. Mr. F. G. Hazell, the general superintendent, welcomes applications for copies of the report from hospital secretaries and interested people.

### Malt and Cod Liver Oil.

In many foods the vitamines are destroyed by cooking and it is therefore necessary to supply the deficiency by some special preparation such as "Edme" malt and cod liver oil, in which only the finest quality malt extract is used in conjunction with guaranteed non-freezing Norwegian cod liver oil. The special process of manufacture makes the oil in this preparation palatable—a point which parents and nurses will much appreciate. It is consequently much used in hospitals and is valuable after influenza, bad colds, and other debilitating ailments. For those who are suffering from digestive troubles "Edme" malt extract without the oil is recommended. Both preparations are obtainable through chemists and stores.

### Future of the V.A.D.

It has been decided to reconstitute the Central Joint Voluntary Aid Detachment Committee, which was formed during the war to administer the Voluntary Aid Detachments. In the light of experience gained during the war, and with a view to the most economical use of *personnel* it is now proposed to call on the Voluntary Aid Detachments to supplement not only the Territorial Army, as before the war, but also the Royal Navy, the Regular Army, and the Royal Air Force; and the intention is that members of the detachments should be enrolled for duty with any of the Services, as may be required. The Central Joint Voluntary Aid Detachment Council, as it is to be called, will include representatives of the Admiralty and War Office, Air Ministry, Territorial County Associations, Order of St. John and the British Red Cross. It will be responsible to the War Office.

### A Hospital Whist Championship.

The funds of the Montgomery County Infirmary have been benefited to the extent of £413 by means of a whist championship, organised by Mr. E. C. Morgan, the secretary of the institution. Twenty-four qualifying competitions were held in various parts of the county, in which over 3,000 competitors took part. A silver challenge cup, given by Mr. H. E. Breese, the chairman of the Board of Management, was provided for the gentlemen champion, and a silver rose bowl, given by Dr. Shearer, the vice-chairman of the board and senior medical officer, was the challenge trophy for ladies. The first lady and gentleman each received a gold watch, and several other valuable prizes were offered. The championship is likely to prove a valuable source of annual income to the infirmary.

### General Nursing Council.

Sir Wilmot Herringham has been chosen as Chairman of the newly-elected General Nursing Council for England and Wales. The following have been appointed members:—Lady Hobhouse and the Hon. Mrs. Nina Louisa Hills (by the Privy Council); Miss A. S. Barratt and Sir Wilmot P. Herringham (by the Board of Education); the Rev. G. B. Cronshaw, Dr. E. W. Goodall, Dr. Bedford Pierce, Dr. R. D. Smedley, and Sir T. Jenner Verrall (by the Ministry of Health). The following have been elected as members of the Council: Miss R. A. Cox-Davies, Miss A. F. Lloyd-Still, Miss E. M. Musson, Miss M. E. Sparshott, Miss H. A. Alsop, Miss C. Seymour Yapp, Miss Ellinor Smith, Miss Geraldine Bremner, Miss D. S. Coode, Miss Gertrude Cowlin, Miss C. C. du Sautoy, Mr. F. W. Stratton, Miss Maude E. Wiese, Mr. Robert Donaldson, Miss A. M. Bushby, and Miss Susan Villiers.

### The Relay Automatic Telephone.

The Relay Automatic Telephone Co., Ltd. (Marconi House, W.C.), has issued an interesting "Souvenir" of the Relay Automatic Telephone Public Exchange at Fleetwood, manufactured and installed by this company for the British Post Office. In 1914 the first "all relay" switchboard to demonstrate the working for 100 lines was brought to this country. The relay system was discovered after considerable development work. While endeavouring to effect economies in the number of switches necessary, a new trunking principle was evolved which it was subsequently seen opened the way for the elimination of electro-mechanical switchgear altogether, and the substitution therefor of simple telephone relays, already largely used in manual telephone exchanges. The system has now been well proved by continual use on innumerable private installations, including many important banks and mercantile houses in London. The Government of India chose the "Relay" system for public use in many towns in India, also for the Government Houses at Bombay and Poona, while the company has also given the first public automatic telephone service in the Union of South Africa.

### Reforms in Lunatic Asylums.

The Board of Control has sent a circular letter to the clerks of the visiting committees urging the importance of carrying out some recommendations made by the committee which investigated the charges of Dr. Lomax last year. These recommendations include the placing of a medical man at the head of each asylum as superintendent, an increase in the number of assistant medical officers, and facilities for study leave; the appointment of consultants and of visiting dental surgeons, the employment of patients capable of being employed, and after-care. The announcement is made that "it is intended to include a clause in the proposed Mental Treatment Bill facilitating the combination of local authorities for any purpose, including research and the provision of properly equipped laboratories." This refers to the suggestion that certain centres should be set aside for special research work. It is added that facilities for the early treatment of incipient mental disease without certification will be included in the Bill.

### Dental Board Lectures.

Under the auspices of the Dental Board four lectures will be delivered in London, Manchester and Edinburgh on "The Diseases of the Periodental Tissues due to Infection in their Relation to Toxaemia." The lecturers and subjects will be as follows:—J. G. Turner, F.R.C.S., L.D.S., The Local Clinical Symptoms; J. Howard Mummery, O.B.E., D.D.Sc. Pen., M.R.C.S., The Pathohistology; Professor E. E. Glynn, M.D., F.R.C.P., The Bacteriology, Sir William Willcox, K.C.I.E., C.B., C.M.G., M.D., F.R.C.P., The Systemic Effects. In London the lectures will be given at Barnes Hall, The Royal Society of Medicine, 1, Wimpole Street, W.1, at 5.30 p.m., on March 1, 8, 15 and 22. The chair will be taken by the Rt. Hon. Francis Dyke Acland, Chairman of the Dental Board of the United Kingdom. In Manchester the lectures will be given at the Physiology Lecture Theatre, Victoria University, at 5.30 p.m., on March 1, 8, 15 and April 6. In Edinburgh they will be delivered in Professor Thomson's Lecture Room at the University, at 5.30 p.m., on March 2, 9, 16 and April 7. The lectures are open to registered dentists and medical practitioners.

